# Supplementary figures and images for: Cytosolic CRISPR RNAs for efficient application of RNA-targeting CRISPR-Cas systems
Source: EMBO Rep. 2025 Feb 26;26(7):1891–912. doi: 10.1038/s44319-025-00399-4 (PMC11976971; doi:10.1038/s44319-025-00399-4)

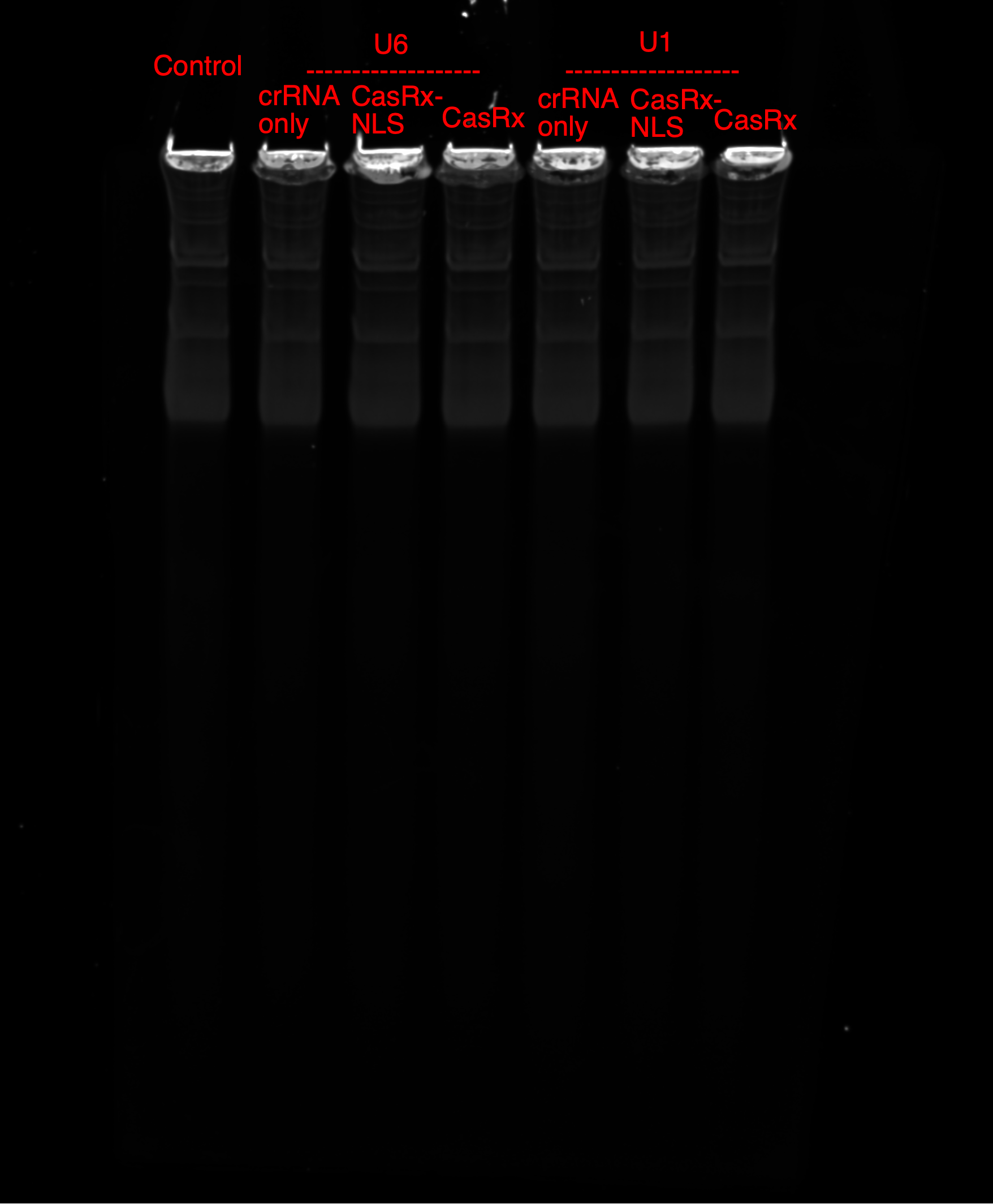

Supplement: Supplementary file 6 — Source data Fig. 1 [file 44319_2025_399_MOESM6_ESM.zip › Figure 1/1D/EtBr staining_CasRx crRNA.tif]

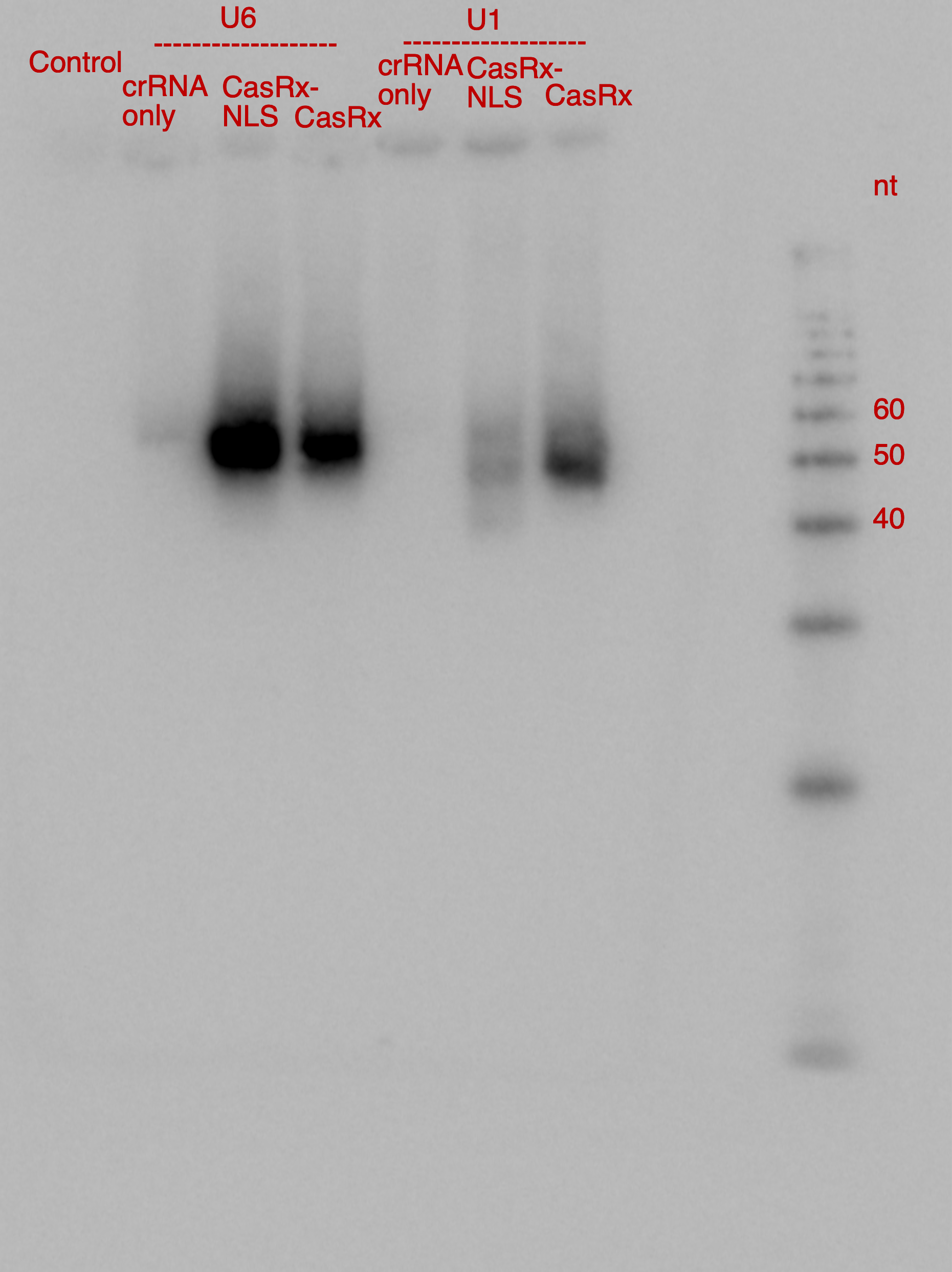

Supplement: Supplementary file 6 — Source data Fig. 1 [file 44319_2025_399_MOESM6_ESM.zip › Figure 1/1D/northern_CasRx crRNA.tif]

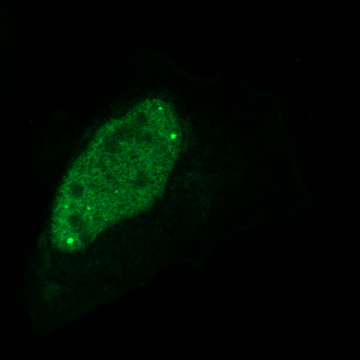

Supplement: Supplementary file 6 — Source data Fig. 1 [file 44319_2025_399_MOESM6_ESM.zip › Figure 1/1C/immunocytochemistry + RNA FISH_U6-driven crRNA + CasRx_ATTO-488.tif]

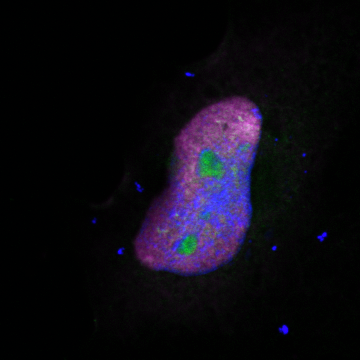

Supplement: Supplementary file 6 — Source data Fig. 1 [file 44319_2025_399_MOESM6_ESM.zip › Figure 1/1C/immunocytochemistry + RNA FISH_U6-driven crRNA + CasRx-NLS_Merge.tif]

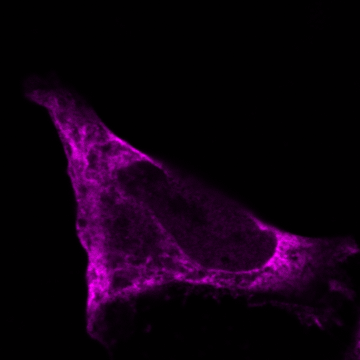

Supplement: Supplementary file 6 — Source data Fig. 1 [file 44319_2025_399_MOESM6_ESM.zip › Figure 1/1C/immunocytochemistry + RNA FISH_U1-driven crRNA + CasRx_HA.tif]

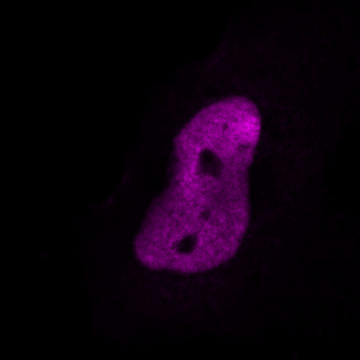

Supplement: Supplementary file 6 — Source data Fig. 1 [file 44319_2025_399_MOESM6_ESM.zip › Figure 1/1C/immunocytochemistry + RNA FISH_U6-driven crRNA + CasRx-NLS_HA.tif]

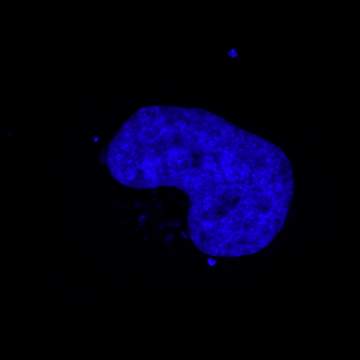

Supplement: Supplementary file 6 — Source data Fig. 1 [file 44319_2025_399_MOESM6_ESM.zip › Figure 1/1C/immunocytochemistry + RNA FISH_U1-driven crRNA + CasRx-NLS_DAPI.tif]

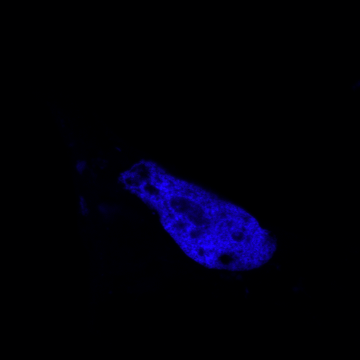

Supplement: Supplementary file 6 — Source data Fig. 1 [file 44319_2025_399_MOESM6_ESM.zip › Figure 1/1C/immunocytochemistry + RNA FISH_U1-driven crRNA + CasRx_DAPI.tif]

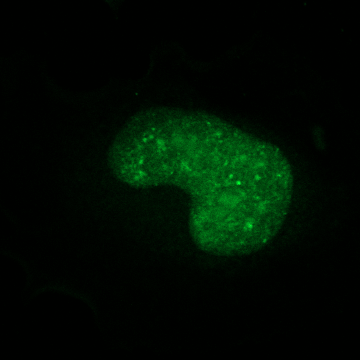

Supplement: Supplementary file 6 — Source data Fig. 1 [file 44319_2025_399_MOESM6_ESM.zip › Figure 1/1C/immunocytochemistry + RNA FISH_U1-driven crRNA + CasRx-NLS_ATTO-488.tif]

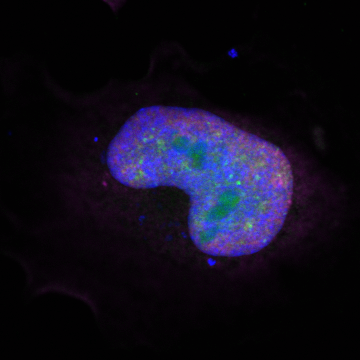

Supplement: Supplementary file 6 — Source data Fig. 1 [file 44319_2025_399_MOESM6_ESM.zip › Figure 1/1C/immunocytochemistry + RNA FISH_U1-driven crRNA + CasRx-NLS_Merge.tif]

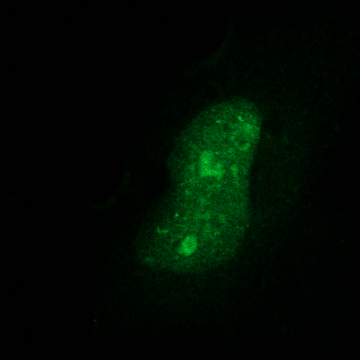

Supplement: Supplementary file 6 — Source data Fig. 1 [file 44319_2025_399_MOESM6_ESM.zip › Figure 1/1C/immunocytochemistry + RNA FISH_U6-driven crRNA + CasRx-NLS_ATTO-488.tif]

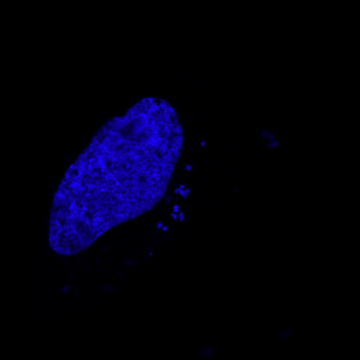

Supplement: Supplementary file 6 — Source data Fig. 1 [file 44319_2025_399_MOESM6_ESM.zip › Figure 1/1C/immunocytochemistry + RNA FISH_U6-driven crRNA + CasRx_DAPI.tif]

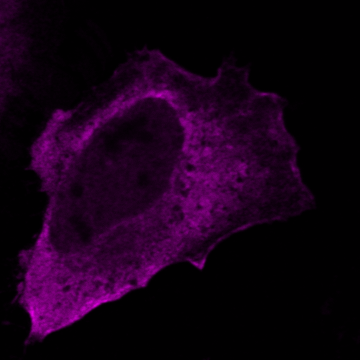

Supplement: Supplementary file 6 — Source data Fig. 1 [file 44319_2025_399_MOESM6_ESM.zip › Figure 1/1C/immunocytochemistry + RNA FISH_U6-driven crRNA + CasRx_HA.tif]

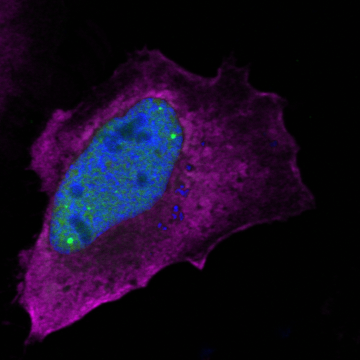

Supplement: Supplementary file 6 — Source data Fig. 1 [file 44319_2025_399_MOESM6_ESM.zip › Figure 1/1C/immunocytochemistry + RNA FISH_U6-driven crRNA + CasRx_Merge.tif]

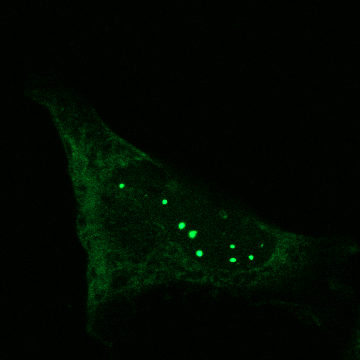

Supplement: Supplementary file 6 — Source data Fig. 1 [file 44319_2025_399_MOESM6_ESM.zip › Figure 1/1C/immunocytochemistry + RNA FISH_U1-driven crRNA + CasRx_ATTO-488.tif]

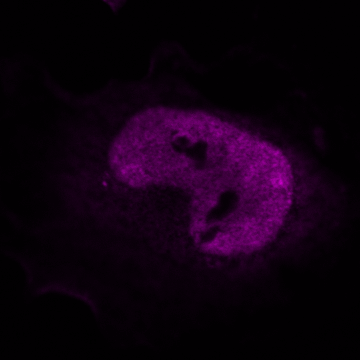

Supplement: Supplementary file 6 — Source data Fig. 1 [file 44319_2025_399_MOESM6_ESM.zip › Figure 1/1C/immunocytochemistry + RNA FISH_U1-driven crRNA + CasRx-NLS_HA.tif]

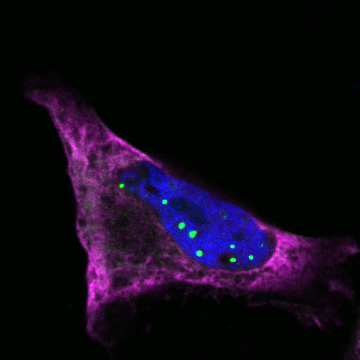

Supplement: Supplementary file 6 — Source data Fig. 1 [file 44319_2025_399_MOESM6_ESM.zip › Figure 1/1C/immunocytochemistry + RNA FISH_U1-driven crRNA + CasRx_Merge.tif]

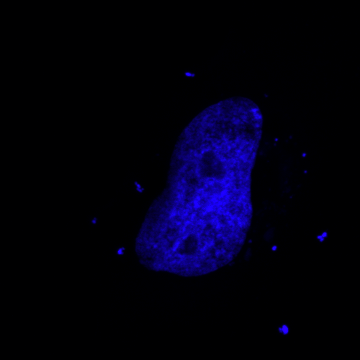

Supplement: Supplementary file 6 — Source data Fig. 1 [file 44319_2025_399_MOESM6_ESM.zip › Figure 1/1C/immunocytochemistry + RNA FISH_U6-driven crRNA + CasRx-NLS_DAPI.tif]

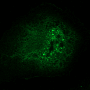

Supplement: Supplementary file 10 — Figure EV Source Data [file 44319_2025_399_MOESM10_ESM.zip › Figure EV2/EV2C/RNA FISH_U1-driven EGFP crRNA + CasRx_ATTO-488.tif]

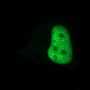

Supplement: Supplementary file 10 — Figure EV Source Data [file 44319_2025_399_MOESM10_ESM.zip › Figure EV2/EV2C/RNA FISH_U6-driven EGFP crRNA + CasRx-NLS_ATTO-488.tif]

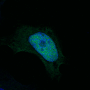

Supplement: Supplementary file 10 — Figure EV Source Data [file 44319_2025_399_MOESM10_ESM.zip › Figure EV2/EV2C/RNA FISH_U6-driven mCherry crRNA + CasRx_Merge.tif]

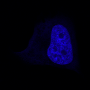

Supplement: Supplementary file 10 — Figure EV Source Data [file 44319_2025_399_MOESM10_ESM.zip › Figure EV2/EV2C/RNA FISH_U6-driven EGFP crRNA + CasRx-NLS_DAPI.tif]

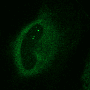

Supplement: Supplementary file 10 — Figure EV Source Data [file 44319_2025_399_MOESM10_ESM.zip › Figure EV2/EV2C/RNA FISH_U1-driven mCherry crRNA + CasRx_ATTO-488.tif]

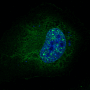

Supplement: Supplementary file 10 — Figure EV Source Data [file 44319_2025_399_MOESM10_ESM.zip › Figure EV2/EV2C/RNA FISH_U1-driven EGFP crRNA + CasRx_Merge.tif]

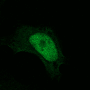

Supplement: Supplementary file 10 — Figure EV Source Data [file 44319_2025_399_MOESM10_ESM.zip › Figure EV2/EV2C/RNA FISH_U6-driven mCherry crRNA + CasRx_ATTO-488.tif]

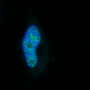

Supplement: Supplementary file 10 — Figure EV Source Data [file 44319_2025_399_MOESM10_ESM.zip › Figure EV2/EV2C/RNA FISH_U6-driven mCherry crRNA + CasRx-NLS_Merge.tif]

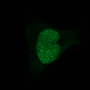

Supplement: Supplementary file 10 — Figure EV Source Data [file 44319_2025_399_MOESM10_ESM.zip › Figure EV2/EV2C/RNA FISH_U1-driven mCherry crRNA + CasRx-NLS_ATTO-488.tif]

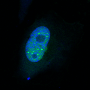

Supplement: Supplementary file 10 — Figure EV Source Data [file 44319_2025_399_MOESM10_ESM.zip › Figure EV2/EV2C/RNA FISH_U1-driven EGFP crRNA + CasRx-NLS_Merge.tif]

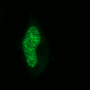

Supplement: Supplementary file 10 — Figure EV Source Data [file 44319_2025_399_MOESM10_ESM.zip › Figure EV2/EV2C/RNA FISH_U6-driven mCherry crRNA + CasRx-NLS_ATTO-488.tif]

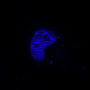

Supplement: Supplementary file 10 — Figure EV Source Data [file 44319_2025_399_MOESM10_ESM.zip › Figure EV2/EV2C/RNA FISH_U6-driven EGFP crRNA + CasRx_DAPI.tif]

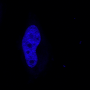

Supplement: Supplementary file 10 — Figure EV Source Data [file 44319_2025_399_MOESM10_ESM.zip › Figure EV2/EV2C/RNA FISH_U6-driven mCherry crRNA + CasRx-NLS_DAPI.tif]

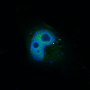

Supplement: Supplementary file 10 — Figure EV Source Data [file 44319_2025_399_MOESM10_ESM.zip › Figure EV2/EV2C/RNA FISH_U6-driven EGFP crRNA + CasRx_Merge.tif]

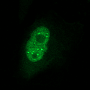

Supplement: Supplementary file 10 — Figure EV Source Data [file 44319_2025_399_MOESM10_ESM.zip › Figure EV2/EV2C/RNA FISH_U1-driven EGFP crRNA + CasRx-NLS_ATTO-488.tif]

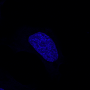

Supplement: Supplementary file 10 — Figure EV Source Data [file 44319_2025_399_MOESM10_ESM.zip › Figure EV2/EV2C/RNA FISH_U6-driven mCherry crRNA + CasRx_DAPI.tif]

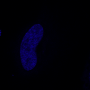

Supplement: Supplementary file 10 — Figure EV Source Data [file 44319_2025_399_MOESM10_ESM.zip › Figure EV2/EV2C/RNA FISH_U1-driven mCherry crRNA + CasRx_DAPI.tif]

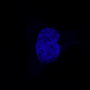

Supplement: Supplementary file 10 — Figure EV Source Data [file 44319_2025_399_MOESM10_ESM.zip › Figure EV2/EV2C/RNA FISH_U1-driven mCherry crRNA + CasRx-NLS_DAPI.tif]

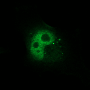

Supplement: Supplementary file 10 — Figure EV Source Data [file 44319_2025_399_MOESM10_ESM.zip › Figure EV2/EV2C/RNA FISH_U6-driven EGFP crRNA + CasRx_ATTO-488.tif]

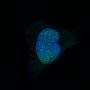

Supplement: Supplementary file 10 — Figure EV Source Data [file 44319_2025_399_MOESM10_ESM.zip › Figure EV2/EV2C/RNA FISH_U1-driven mCherry crRNA + CasRx-NLS_Merge.tif]

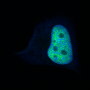

Supplement: Supplementary file 10 — Figure EV Source Data [file 44319_2025_399_MOESM10_ESM.zip › Figure EV2/EV2C/RNA FISH_U6-driven EGFP crRNA + CasRx-NLS_Merge.tif]

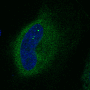

Supplement: Supplementary file 10 — Figure EV Source Data [file 44319_2025_399_MOESM10_ESM.zip › Figure EV2/EV2C/RNA FISH_U1-driven mCherry crRNA + CasRx_Merge.tif]

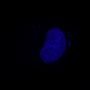

Supplement: Supplementary file 10 — Figure EV Source Data [file 44319_2025_399_MOESM10_ESM.zip › Figure EV2/EV2C/RNA FISH_U1-driven EGFP crRNA + CasRx_DAPI.tif]

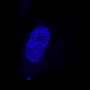

Supplement: Supplementary file 10 — Figure EV Source Data [file 44319_2025_399_MOESM10_ESM.zip › Figure EV2/EV2C/RNA FISH_U1-driven EGFP crRNA + CasRx-NLS_DAPI.tif]

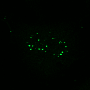

Supplement: Supplementary file 10 — Figure EV Source Data [file 44319_2025_399_MOESM10_ESM.zip › Figure EV2/EV2A_bottom/RNA FISH_U1 crRNA_ATTO-488.tif]

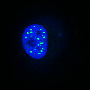

Supplement: Supplementary file 10 — Figure EV Source Data [file 44319_2025_399_MOESM10_ESM.zip › Figure EV2/EV2A_bottom/RNA FISH_U6 crRNA_Merge.tif]

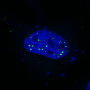

Supplement: Supplementary file 10 — Figure EV Source Data [file 44319_2025_399_MOESM10_ESM.zip › Figure EV2/EV2A_bottom/RNA FISH_U1 crRNA_Merge.tif]

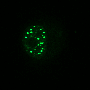

Supplement: Supplementary file 10 — Figure EV Source Data [file 44319_2025_399_MOESM10_ESM.zip › Figure EV2/EV2A_bottom/RNA FISH_U6 crRNA_ATTO-488.tif]

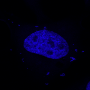

Supplement: Supplementary file 10 — Figure EV Source Data [file 44319_2025_399_MOESM10_ESM.zip › Figure EV2/EV2A_bottom/RNA FISH_U1 crRNA_DAPI.tif]

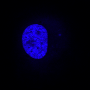

Supplement: Supplementary file 10 — Figure EV Source Data [file 44319_2025_399_MOESM10_ESM.zip › Figure EV2/EV2A_bottom/RNA FISH_U6 crRNA_DAPI.tif]

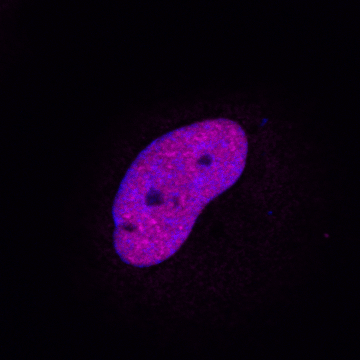

Supplement: Supplementary file 10 — Figure EV Source Data [file 44319_2025_399_MOESM10_ESM.zip › Figure EV2/EV2A_top/immunocytochemistry_CasRx-NLS_Merge.tif]

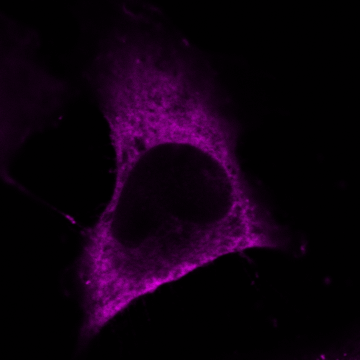

Supplement: Supplementary file 10 — Figure EV Source Data [file 44319_2025_399_MOESM10_ESM.zip › Figure EV2/EV2A_top/immunocytochemistry_CasRx_HA.tif]

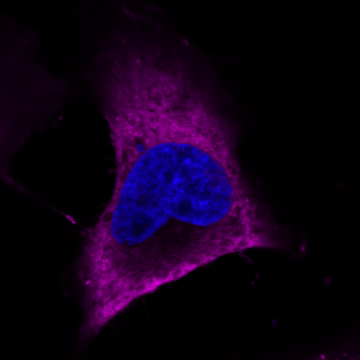

Supplement: Supplementary file 10 — Figure EV Source Data [file 44319_2025_399_MOESM10_ESM.zip › Figure EV2/EV2A_top/immunocytochemistry_CasRx_Merge.tif]

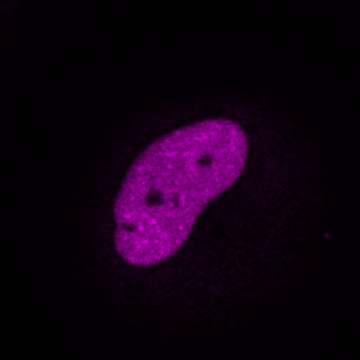

Supplement: Supplementary file 10 — Figure EV Source Data [file 44319_2025_399_MOESM10_ESM.zip › Figure EV2/EV2A_top/immunocytochemistry_CasRx-NLS_HA.tif]

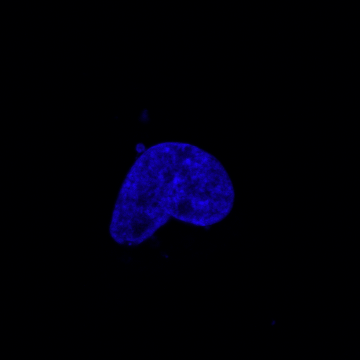

Supplement: Supplementary file 10 — Figure EV Source Data [file 44319_2025_399_MOESM10_ESM.zip › Figure EV2/EV2A_top/immunocytochemistry_CasRx_DAPI.tif]

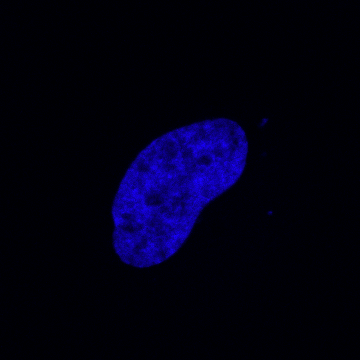

Supplement: Supplementary file 10 — Figure EV Source Data [file 44319_2025_399_MOESM10_ESM.zip › Figure EV2/EV2A_top/immunocytochemistry_CasRx-NLS_DAPI.tif]

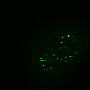

Supplement: Supplementary file 10 — Figure EV Source Data [file 44319_2025_399_MOESM10_ESM.zip › Figure EV2/EV2A_middle/RNA FISH_U1 crRNA_ATTO-488.tif]

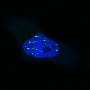

Supplement: Supplementary file 10 — Figure EV Source Data [file 44319_2025_399_MOESM10_ESM.zip › Figure EV2/EV2A_middle/RNA FISH_U6 crRNA_Merge.tif]

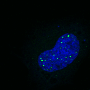

Supplement: Supplementary file 10 — Figure EV Source Data [file 44319_2025_399_MOESM10_ESM.zip › Figure EV2/EV2A_middle/RNA FISH_U1 crRNA_Merge.tif]

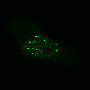

Supplement: Supplementary file 10 — Figure EV Source Data [file 44319_2025_399_MOESM10_ESM.zip › Figure EV2/EV2A_middle/RNA FISH_U6 crRNA_ATTO-488.tif]

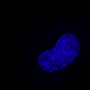

Supplement: Supplementary file 10 — Figure EV Source Data [file 44319_2025_399_MOESM10_ESM.zip › Figure EV2/EV2A_middle/RNA FISH_U1 crRNA_DAPI.tif]

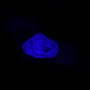

Supplement: Supplementary file 10 — Figure EV Source Data [file 44319_2025_399_MOESM10_ESM.zip › Figure EV2/EV2A_middle/RNA FISH_U6 crRNA_DAPI.tif]

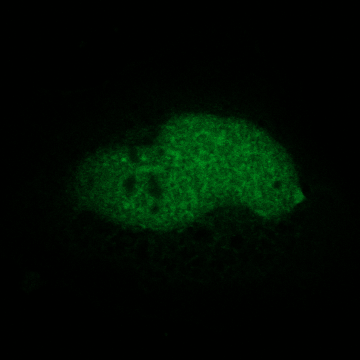

Supplement: Supplementary file 10 — Figure EV Source Data [file 44319_2025_399_MOESM10_ESM.zip › Figure EV3/EV3A/immunocytochemistry + RNA FISH_U6-driven crRNA + CasRx_ATTO-488.tif]

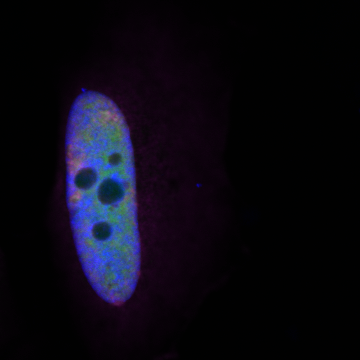

Supplement: Supplementary file 10 — Figure EV Source Data [file 44319_2025_399_MOESM10_ESM.zip › Figure EV3/EV3A/immunocytochemistry + RNA FISH_U6-driven crRNA + CasRx-NLS_Merge.tif]

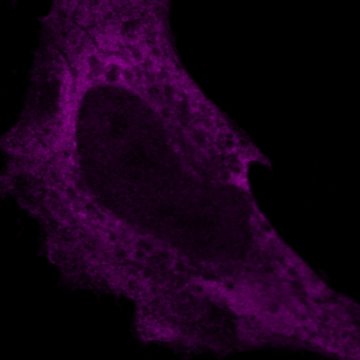

Supplement: Supplementary file 10 — Figure EV Source Data [file 44319_2025_399_MOESM10_ESM.zip › Figure EV3/EV3A/immunocytochemistry + RNA FISH_U1-driven crRNA + CasRx_HA.tif]

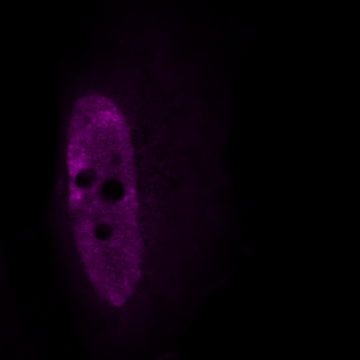

Supplement: Supplementary file 10 — Figure EV Source Data [file 44319_2025_399_MOESM10_ESM.zip › Figure EV3/EV3A/immunocytochemistry + RNA FISH_U6-driven crRNA + CasRx-NLS_HA.tif]

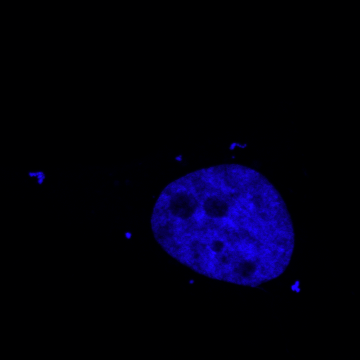

Supplement: Supplementary file 10 — Figure EV Source Data [file 44319_2025_399_MOESM10_ESM.zip › Figure EV3/EV3A/immunocytochemistry + RNA FISH_U1-driven crRNA + CasRx-NLS_DAPI.tif]

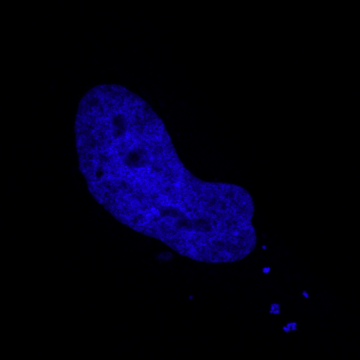

Supplement: Supplementary file 10 — Figure EV Source Data [file 44319_2025_399_MOESM10_ESM.zip › Figure EV3/EV3A/immunocytochemistry + RNA FISH_U1-driven crRNA + CasRx_DAPI.tif]

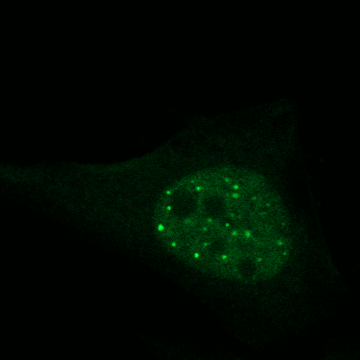

Supplement: Supplementary file 10 — Figure EV Source Data [file 44319_2025_399_MOESM10_ESM.zip › Figure EV3/EV3A/immunocytochemistry + RNA FISH_U1-driven crRNA + CasRx-NLS_ATTO-488.tif]

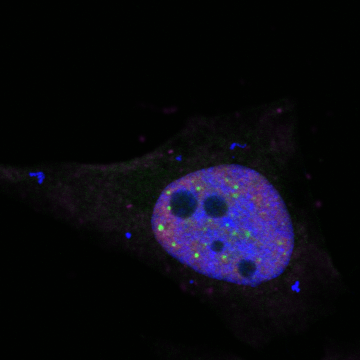

Supplement: Supplementary file 10 — Figure EV Source Data [file 44319_2025_399_MOESM10_ESM.zip › Figure EV3/EV3A/immunocytochemistry + RNA FISH_U1-driven crRNA + CasRx-NLS_Merge.tif]

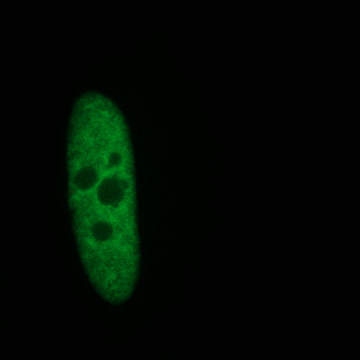

Supplement: Supplementary file 10 — Figure EV Source Data [file 44319_2025_399_MOESM10_ESM.zip › Figure EV3/EV3A/immunocytochemistry + RNA FISH_U6-driven crRNA + CasRx-NLS_ATTO-488.tif]

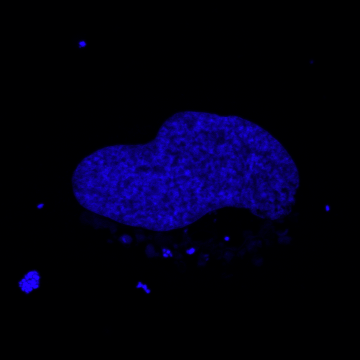

Supplement: Supplementary file 10 — Figure EV Source Data [file 44319_2025_399_MOESM10_ESM.zip › Figure EV3/EV3A/immunocytochemistry + RNA FISH_U6-driven crRNA + CasRx_DAPI.tif]

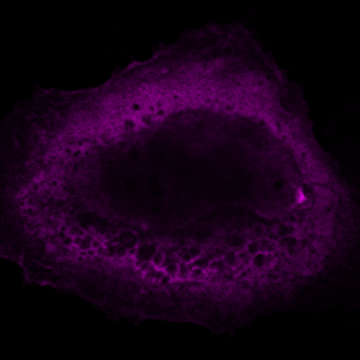

Supplement: Supplementary file 10 — Figure EV Source Data [file 44319_2025_399_MOESM10_ESM.zip › Figure EV3/EV3A/immunocytochemistry + RNA FISH_U6-driven crRNA + CasRx_HA.tif]

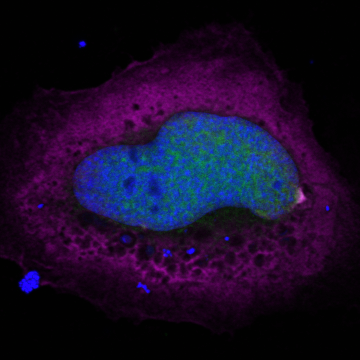

Supplement: Supplementary file 10 — Figure EV Source Data [file 44319_2025_399_MOESM10_ESM.zip › Figure EV3/EV3A/immunocytochemistry + RNA FISH_U6-driven crRNA + CasRx_Merge.tif]

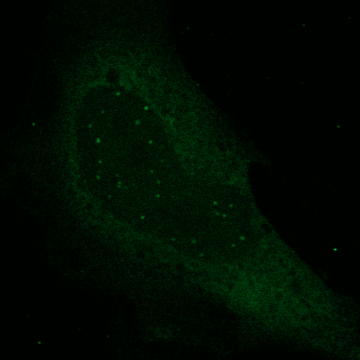

Supplement: Supplementary file 10 — Figure EV Source Data [file 44319_2025_399_MOESM10_ESM.zip › Figure EV3/EV3A/immunocytochemistry + RNA FISH_U1-driven crRNA + CasRx_ATTO-488.tif]

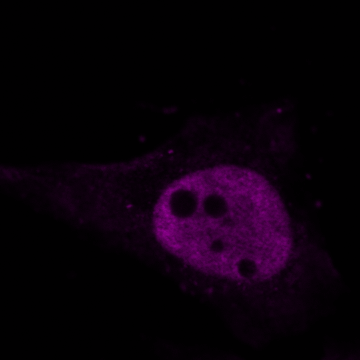

Supplement: Supplementary file 10 — Figure EV Source Data [file 44319_2025_399_MOESM10_ESM.zip › Figure EV3/EV3A/immunocytochemistry + RNA FISH_U1-driven crRNA + CasRx-NLS_HA.tif]

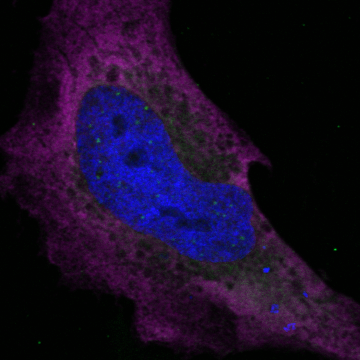

Supplement: Supplementary file 10 — Figure EV Source Data [file 44319_2025_399_MOESM10_ESM.zip › Figure EV3/EV3A/immunocytochemistry + RNA FISH_U1-driven crRNA + CasRx_Merge.tif]

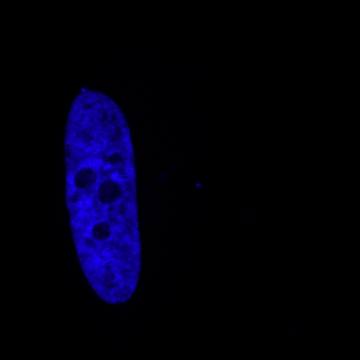

Supplement: Supplementary file 10 — Figure EV Source Data [file 44319_2025_399_MOESM10_ESM.zip › Figure EV3/EV3A/immunocytochemistry + RNA FISH_U6-driven crRNA + CasRx-NLS_DAPI.tif]

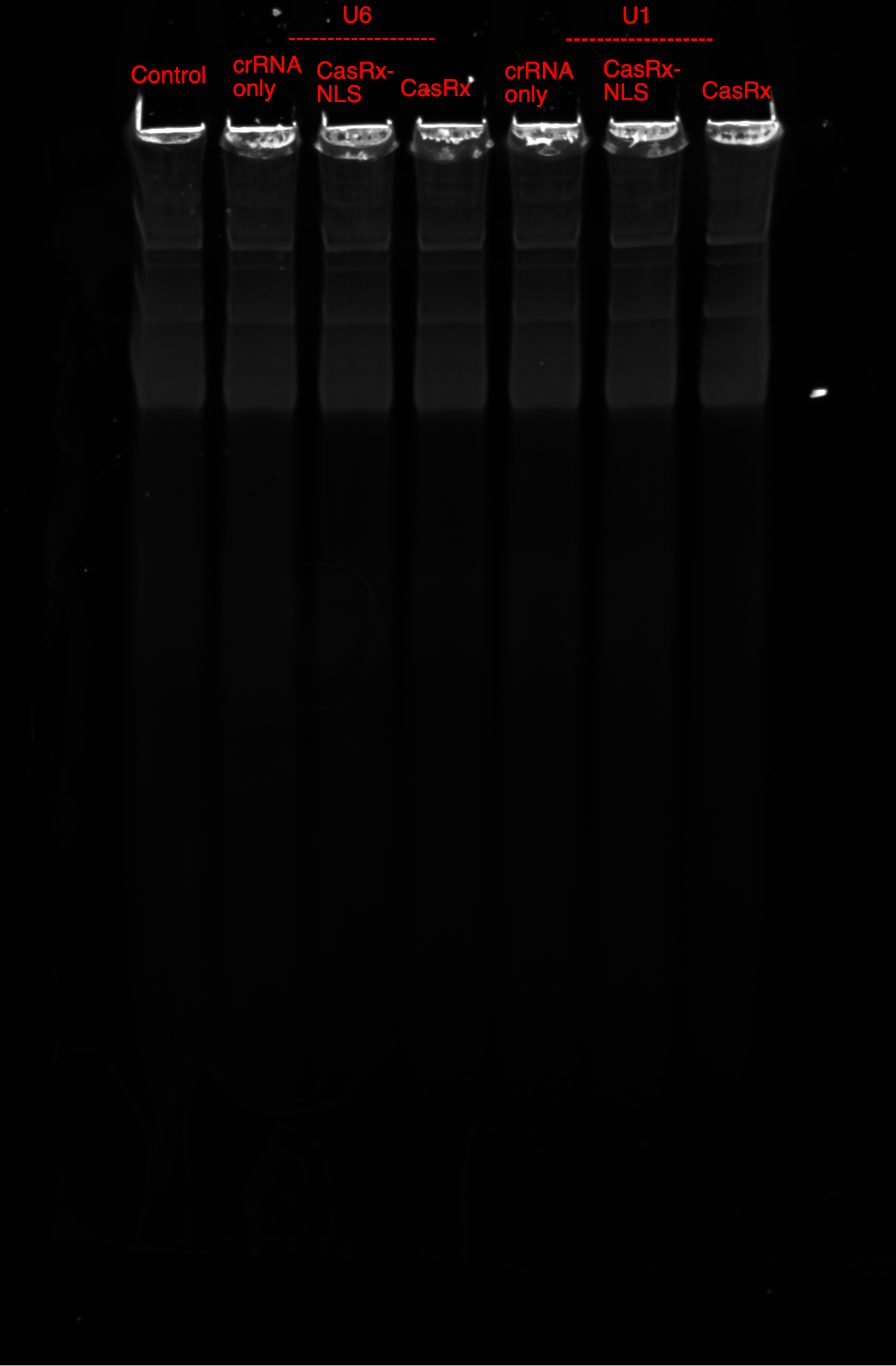

Supplement: Supplementary file 10 — Figure EV Source Data [file 44319_2025_399_MOESM10_ESM.zip › Figure EV3/EV3B/EtBr staining_CasRx crRNA replicate 3.tif]

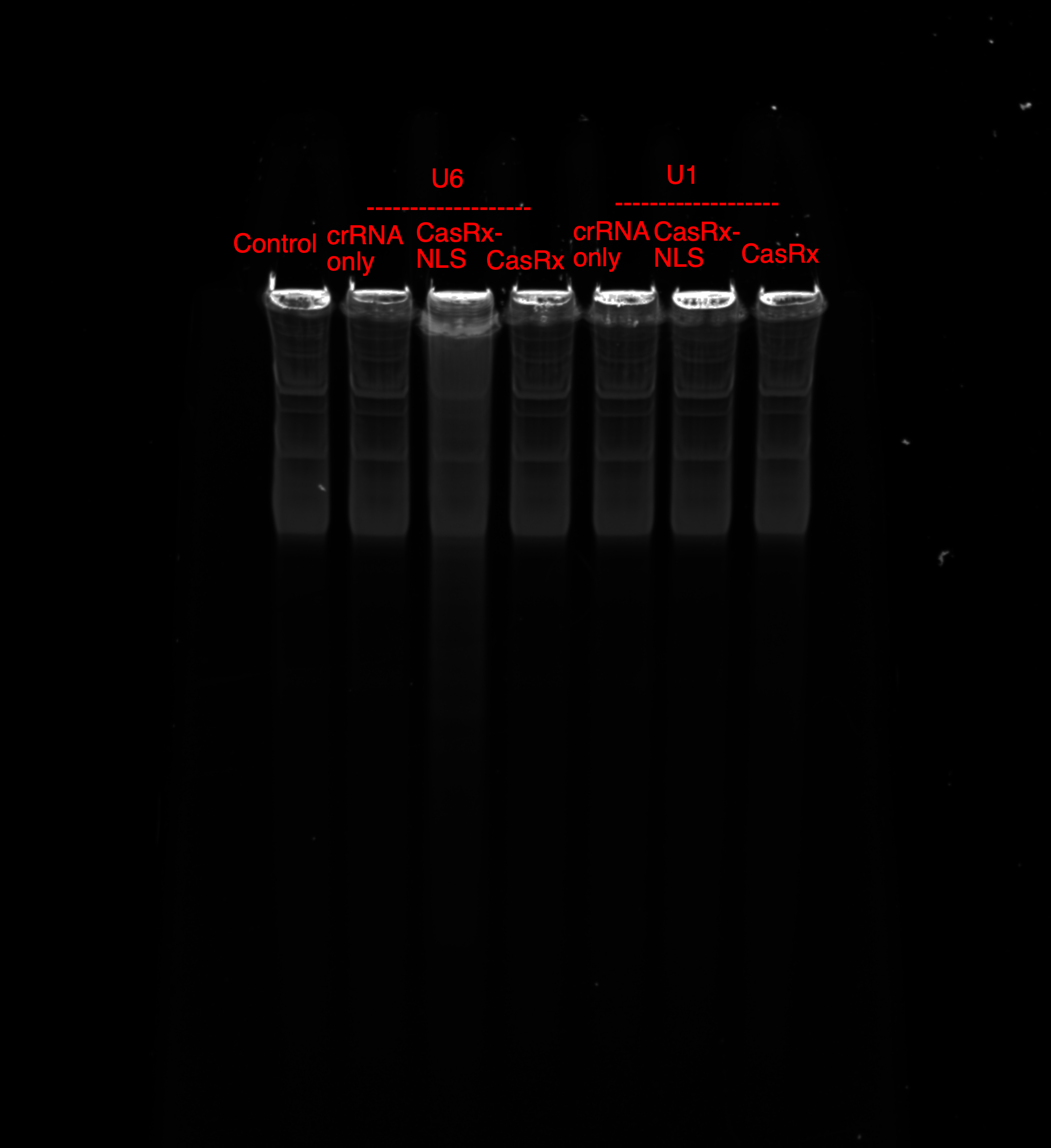

Supplement: Supplementary file 10 — Figure EV Source Data [file 44319_2025_399_MOESM10_ESM.zip › Figure EV3/EV3B/EtBr staining_CasRx crRNA replicate 2.tif]

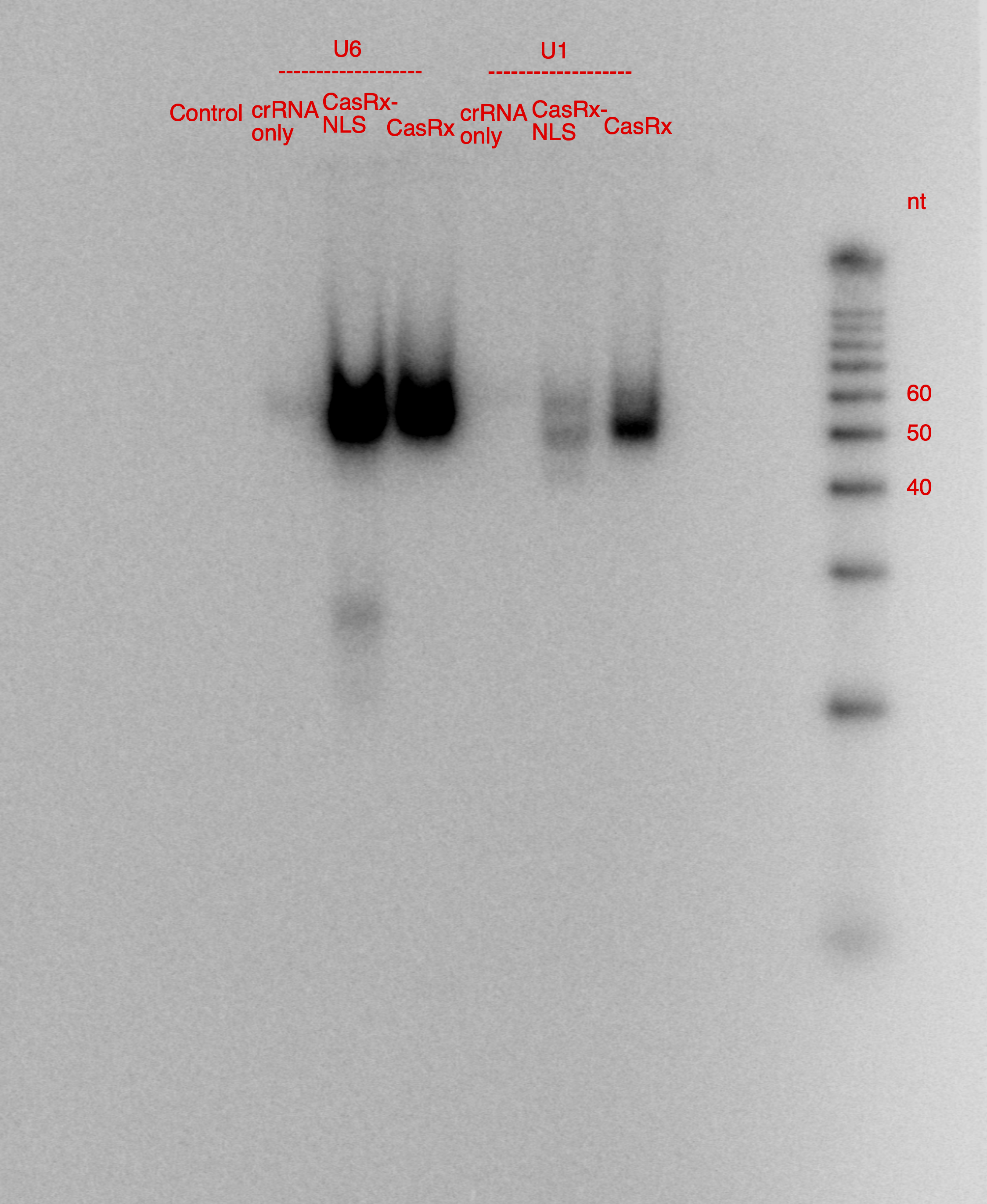

Supplement: Supplementary file 10 — Figure EV Source Data [file 44319_2025_399_MOESM10_ESM.zip › Figure EV3/EV3B/northern_CasRx crRNA replicate 2.tif]

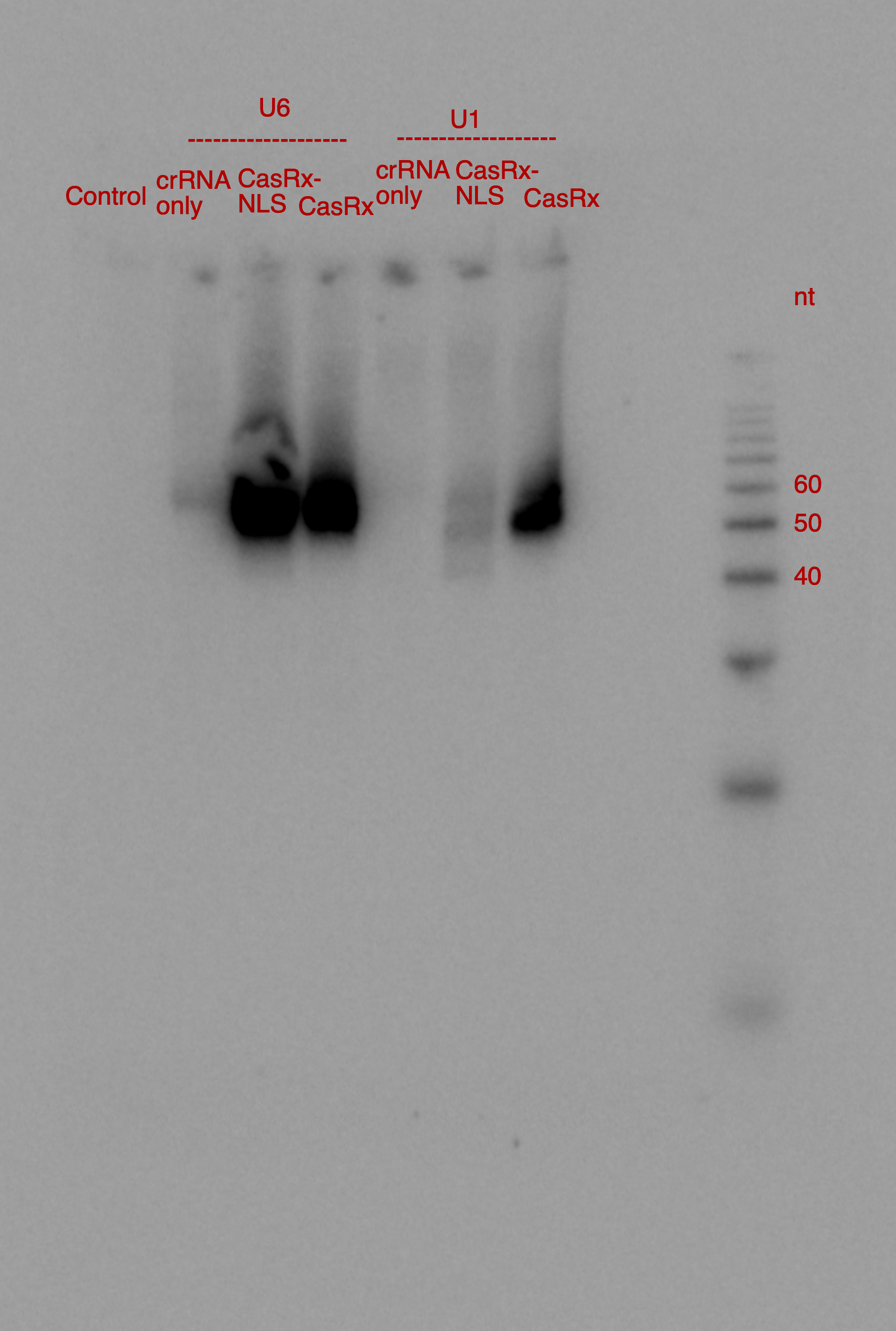

Supplement: Supplementary file 10 — Figure EV Source Data [file 44319_2025_399_MOESM10_ESM.zip › Figure EV3/EV3B/northern_CasRx crRNA replicate 3.tif]

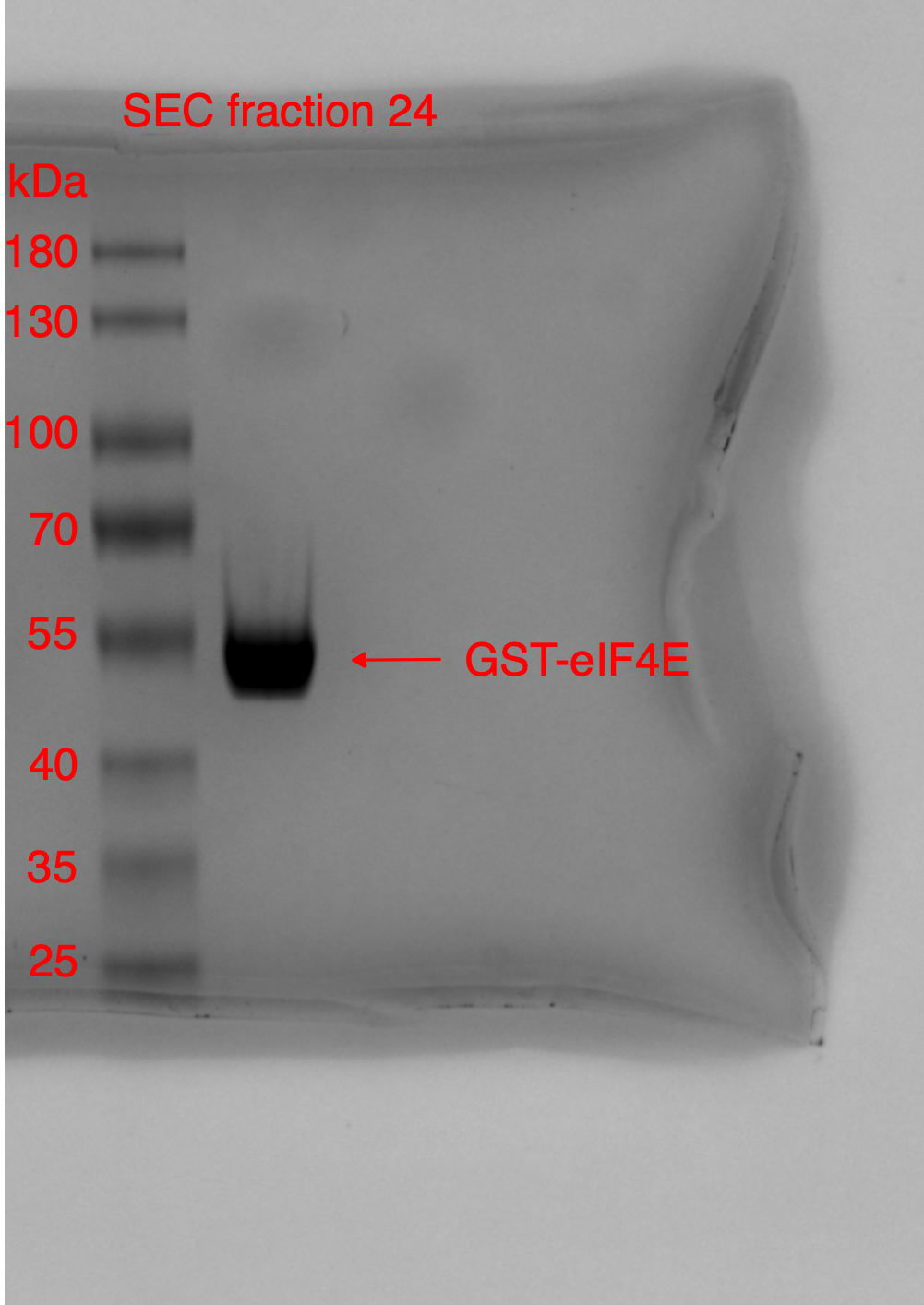

Supplement: Supplementary file 10 — Figure EV Source Data [file 44319_2025_399_MOESM10_ESM.zip › Figure EV4/EV4F/SDS-PAGE_GST-eIF4E.tif]
