# Supplementary material for: Cytosolic CRISPR RNAs for efficient application of RNA-targeting CRISPR-Cas systems
Source: EMBO Rep. 2025 Feb 26;26(7):1891–912. doi: 10.1038/s44319-025-00399-4 (PMC11976971; doi:10.1038/s44319-025-00399-4)
Supplement: Supplementary file 11 — Expanded View Figures [file 44319_2025_399_MOESM11_ESM.pdf]

Expanded View Figures

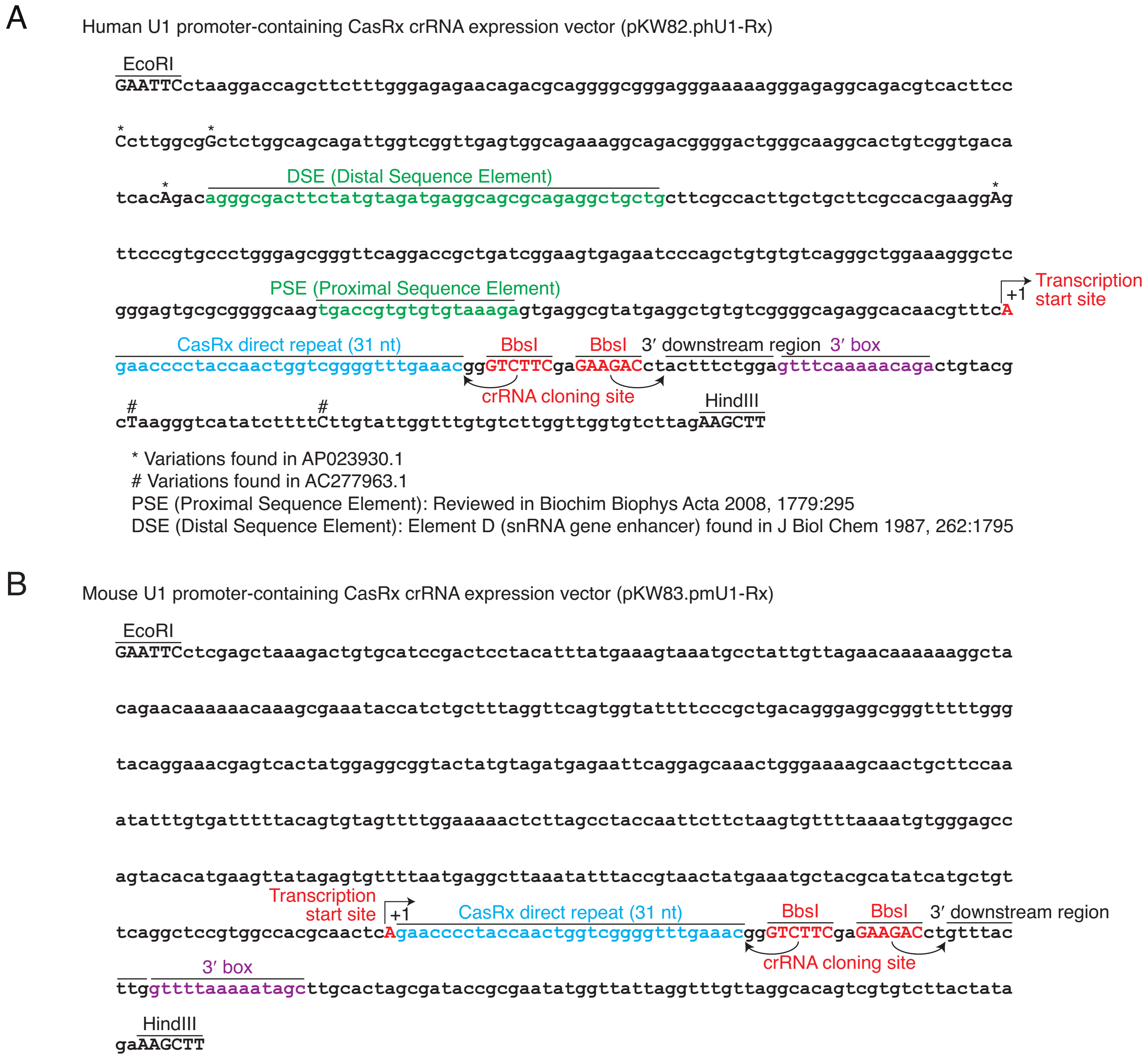

Figure EV1. U1 promoter sequences for cytosolic crRNA.

(**A**) Map of the human U1-driven CasRx crRNA backbone. (**B**) Map of the mouse U1-driven CasRx crRNA backbone.

 © The Author(s)

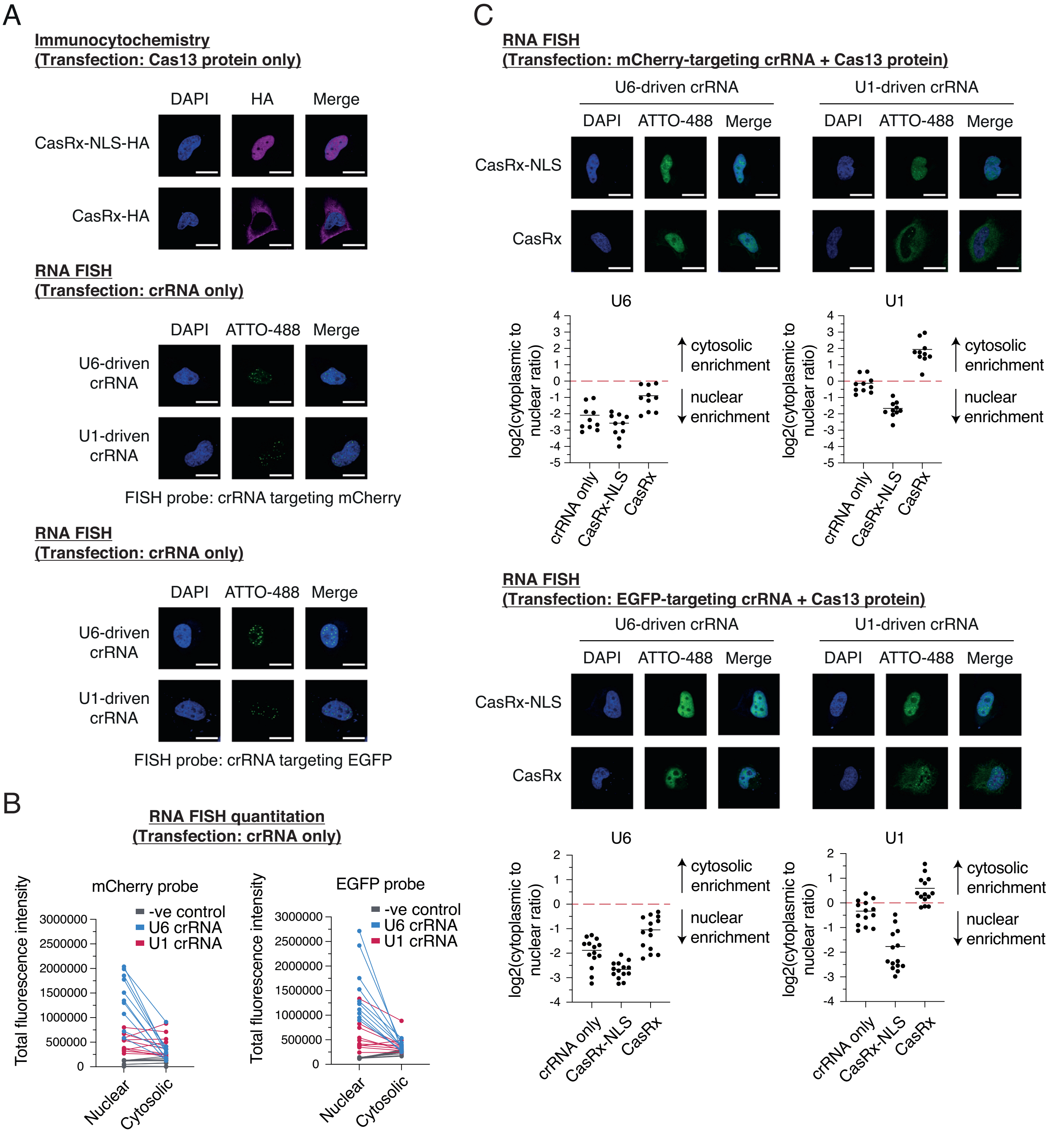

Figure EV2. Repurposing U1 promoter for cytosolic crRNA.

(**A**) Subcellular localization of crRNA and CasRx effector in the absence of the binding partner. Top, immunocytochemistry of CasRx-NLS and CasRx tagged with a HA tag. Middle and bottom, RNA FISH of crRNA. Nuclei were stained with DAPI. CasRx effector was stained with anti-HA antibody. crRNAs targeting mCherry and EGFP were visualized with ATTO-488-conjugated probes. Note that EGFP and mCherry were not expressed and acted as non-human sequences. Scale bars: 20 μm. (**B**) Quantitation of RNA FISH. crRNA signals in the absence of the CasRx protein are shown. $n = 10$ from two biological replicates. (**C**) Subcellular localization of crRNA in the presence of the CasRx protein. Top, crRNA targeting mCherry ($n = 10$ from two biological replicates). Bottom, crRNA targeting EGFP ($n = 14$ from two biological replicates). Note that mCherry and EGFP were not expressed and acted as non-human sequences. Total fluorescence intensity is shown as a scatter plot. Mean is indicated as a horizontal line. Scale bars: 20 μm. Source data are available online for this figure.

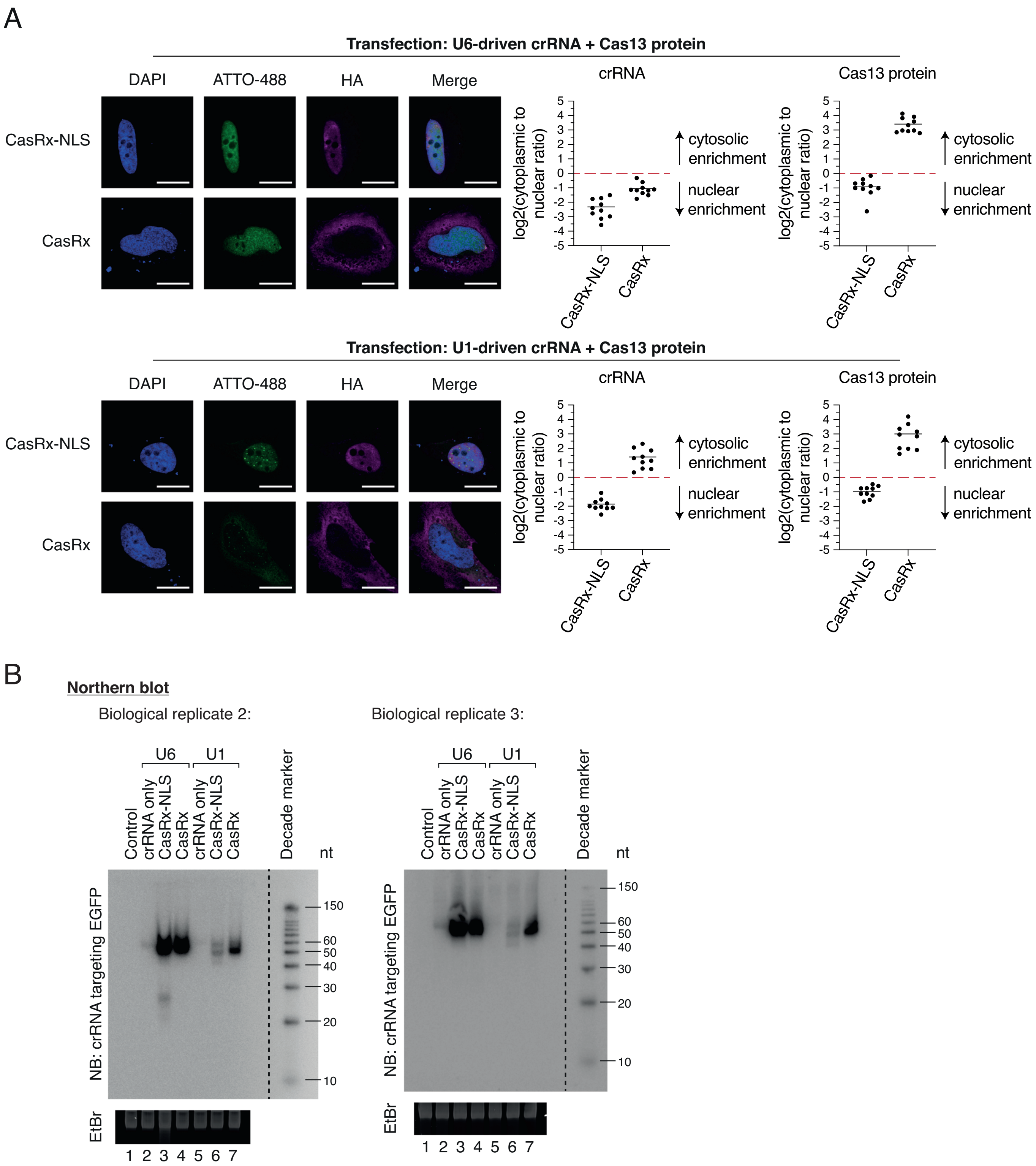

Figure EV3. Subcellular localization of crRNA targeting EGFP.

(**A**) Subcellular localization of crRNA targeting EGFP in the presence of the CasRx protein. RNA FISH was performed following immunocytochemistry of the same cells. Note that EGFP was not expressed and acted as a non-human sequence. Nuclei were stained with DAPI. crRNA targeting EGFP was visualized with an ATTO-488-conjugated probe. CasRx effector was stained with anti-HA antibody. Total fluorescence intensity is shown as a scatter plot. Mean is indicated as a horizontal line. $n = 10$ from three biological replicates. Scale bars: 20 μm. (**B**) Northern blot replicates showing crRNA expression and length distribution. crRNA targeting EGFP was visualized with a 32P-labeled probe. Note that EGFP was not expressed and acted as a non-human sequence. Source data are available online for this figure.

 © The Author(s)

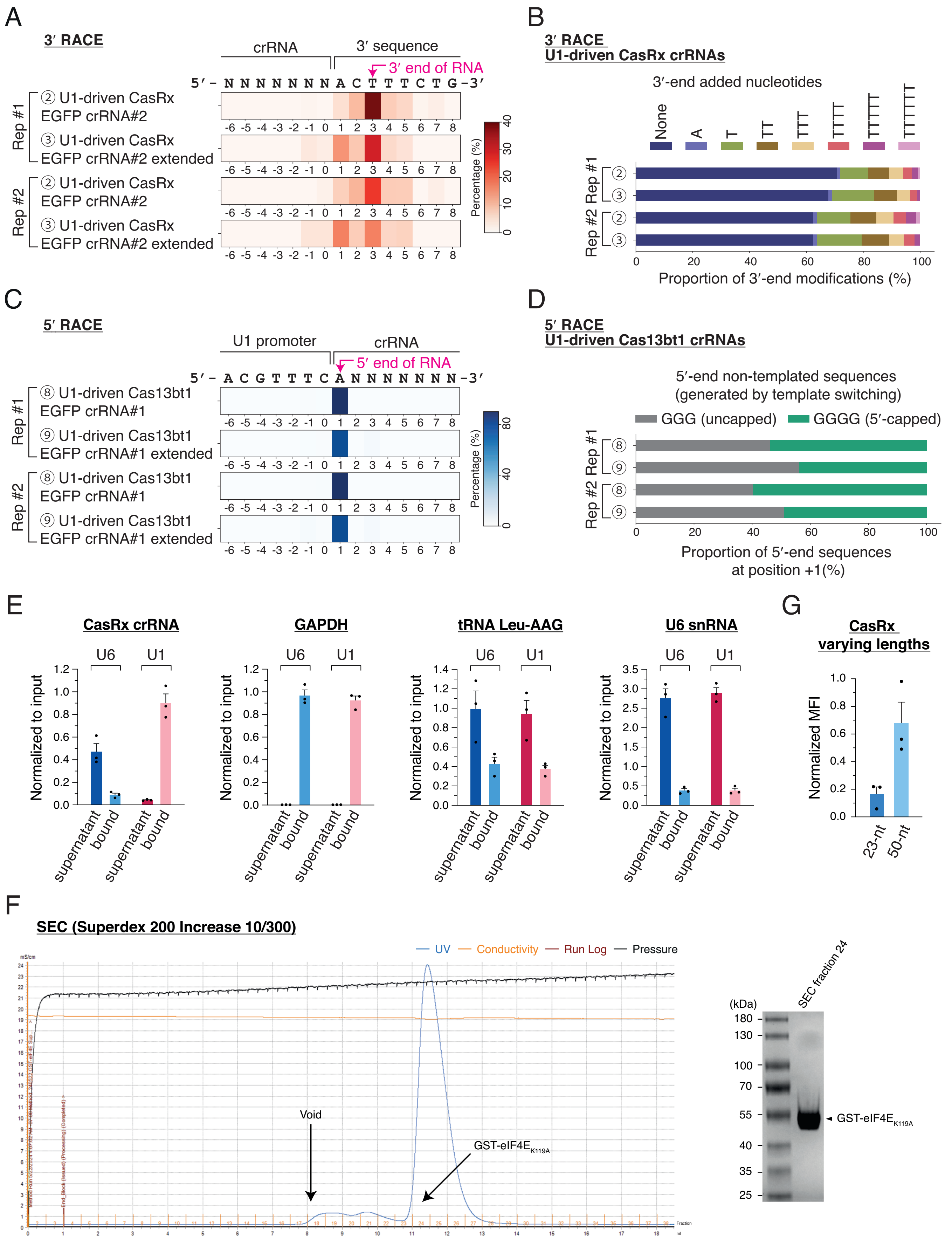
A
3′ RACE
crRNA
3′ sequence
3′ end of RNA
5′- N N N N N N N A C T T T C T G -3′
Rep #1
② U1-driven CasRx EGFP crRNA#2
③ U1-driven CasRx EGFP crRNA#2 extended
Rep #2
② U1-driven CasRx EGFP crRNA#2
③ U1-driven CasRx EGFP crRNA#2 extended
-6 -5 -4 -3 -2 -1 0 1 2 3 4 5 6 7 8
Percentage (%)
B
3′ RACE
U1-driven CasRx crRNAs
3′-end added nucleotides
None A T TT TTT TTTT TTTTT TTTTTT
Proportion of 3′-end modifications (%)
C
5′ RACE
U1 promoter
crRNA
5′ end of RNA
5′- A C G T T T C A N N N N N N N N -3′
⑧ U1-driven Cas13bt1 EGFP crRNA#1
⑨ U1-driven Cas13bt1 EGFP crRNA#1 extended
D
5′ RACE
U1-driven Cas13bt1 crRNAs
5′-end non-templated sequences (generated by template switching)
GGG (uncapped)
GGGG (5′-capped)
Proportion of 5′-end sequences at position +1(%)
E
CasRx crRNA
GAPDH
tRNA Leu-AAG
U6 snRNA
U6
U1
Normalized to input
supernatant
bound
G
CasRx varying lengths
Normalized MFI
23-nt
50-nt
F
SEC (Superdex 200 Increase 10/300)
UV
Conductivity
Run Log
Pressure
Void
GST-eIF4E K119A
SEC fraction 24
(kDa)
180
130
100
70
55
40
35
25
GST-eIF4E K119A

© The Author(s)

Figure EV4. Reproducibility of 5′ RACE and 3′ RACE experiments.

(**A**) Analysis of 3′ end positions. The proportion of each 3′ end position is shown as a heatmap. (**B**) Analysis of 3′ end modifications. (**C**) Analysis of 5′ end positions. The proportion of each 5′ end position is shown as a heatmap. (**D**) Analysis of 5′ end non-templated sequences. (**E**) qRT-PCR following pull-down assay of capped RNA with recombinant mouse $eIF4E_{K119A}$ protein. Enrichment of U1-driven CasRx crRNA in the bound fraction is compared with U6-driven CasRx crRNA. GAPDH mRNA is included to represent capped RNA, while tRNA Leu-AGG and endogenous U6 snRNA are included to represent non-capped RNA. Mean and s.e.m. are shown ($n = 3$, biological triplicates). (**F**) Purification of GST-tagged $eIF4E_{K119A}$. Left, Size-exclusion chromatography (SEC). Right, SDS-PAGE showing homogeneity of the purified GST-tagged $eIF4E_{K119A}$. (**G**) EGFP knockdown by CasRx with varying U1-driven crRNA lengths. Median fluorescence intensity (MFI) is shown. Mean and s.e.m. are shown ($n = 3$, biological triplicates). Source data are available online for this figure.

 © The Author(s)

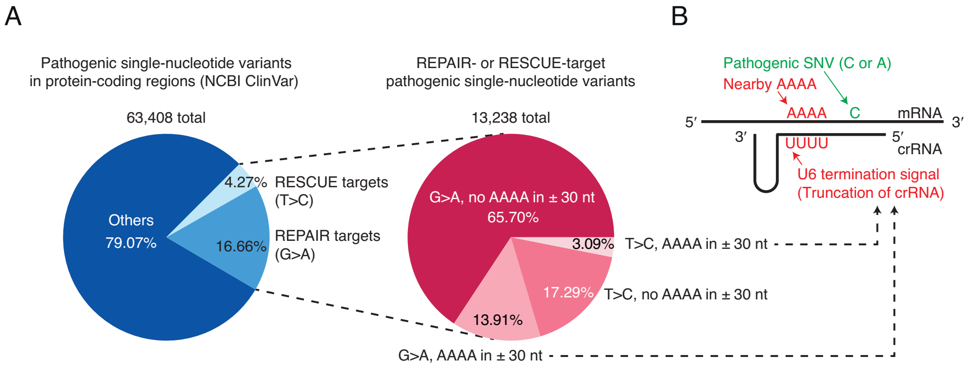

Figure EV5. Targetable pathogenic SNVs (single-nucleotide variants) for REPAIR and RESCUE.

(**A**) ClinVar database was screened for pathogenic SNV that could be targeted by the REPAIR and RESCUE RNA-editing systems. (**B**) Association of the targetable SNVs with adjacent AAAA sequences.

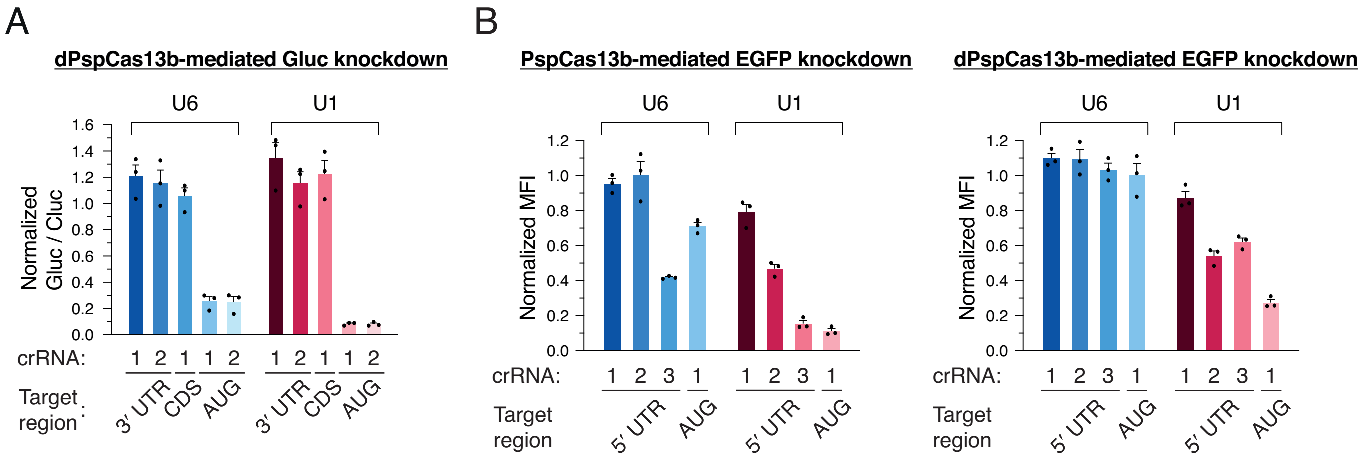

Figure EV6. Enhancing translational repression using U1-driven crRNA.

(**A**) Translational repression of *Gaussia* luciferase using AUG-targeting crRNA and catalytically dead PspCas13b. Mean and s.e.m. are shown ($n = 3$, biological triplicates). (**B**) Translational repression of EGFP using 5′ UTR-targeting crRNA and catalytically dead PspCas13b. Repression efficiency with U1-driven crRNA is compared with the efficiency with U6-driven crRNA. Median fluorescence intensity (MFI) is normalized to the non-targeting control. Mean and s.e.m. are shown ($n = 3$, biological triplicates). Source data are available online for this figure.

 © The Author(s)
